# Prevalence of mild hyponatremia and its association with falls in older adults admitted to an emergency geriatric medicine unit (the MUPA unit)

**DOI:** 10.1186/s12877-019-1282-0

**Published:** 2019-10-15

**Authors:** Sophie Boyer, Caroline Gayot, Charlotte Bimou, Thomas Mergans, Patrick Kajeu, Muriel Castelli, Thierry Dantoine, Achille Tchalla

**Affiliations:** 1 EA 6310 HAVAE Handicap Activité Vieillissement Autonomie Environnement, Université de Limoges, F-8705 Limoges, France; 2 Unité de Recherche Clinique et Innovation (URCI) en Gérontologie (Axe Silver économie, e-santé et Télémédecine), Pôle HU Gérontologie Clinique, CHU de Limoges, 2 Avenue Martin-Luther King, 87042 Limoges, France; 3 CHU de Limoges, Pôle HU Gérontologie Clinique, 2 Avenue Martin-Luther King, F-87042 Limoges, France; 4 Unité de Médecine d’Urgence de la Personne Âgée, Pôle Gérontologie Clinique, Centre Hospitalier Universitaire de Limoges, 2 Avenue Martin-Luther King, F-87042 Limoges, France; 5 Geriatric Medicine, University of Limoges CHU Limoges, IFR 145 GEIST, EA 6310 HAVAE (Handicap Activité Vieillissement Autonomie et Environnement), F-87025 Limoges, France

**Keywords:** Mild hyponatremia, Falls, Emergency department, Older adult, Prevalence, Prevention

## Abstract

**Background:**

Hyponatremia is the most common electrolyte disorder in older adults and it can increase morbidity and mortality. Approximately one in three older adults fall each year; mild chronic hyponatremia can predispose this group to injurious falls and fractures and serum levels of sodium can also influence bone health. Little is known regarding the association between mild chronic hyponatremia and injurious fall prevalence in elderly patients admitted to the Emergency Department (ED). Therefore, the present study investigated the link between mild hyponatremia and the risk of injurious falls in elderly patients admitted to the Emergency Geriatric Medicine Unit (The MUPA Unit).

**Methods:**

This cross-sectional study was conducted over 4 months and included patients ≥75 years of age who were admitted to the MUPA Unit of University Hospital Center of Limoges (France). Sociodemographic factors, fall events, comorbidities, medications, and sodium levels were assessed (hyponatremia was considered as sodium level < 136 mEq/L). Additionally, the short Comprehensive Geriatric Assessment (short-CGA), the Frailty score on the Short Emergency Geriatric Assessment (SEGA), and the Katz Activity of Daily Living (ADL) scale were administered.

**Results:**

Of the 696 cases included in the final analysis, the mean age was 86.1 ± 5.6 years and 63.1% were female. The prevalence of falls was 27.9% (95% confidence interval [CI]: 24.6–31.2%) and that of mild hyponatremia was 15.9% (95% CI: 13.2–18.6%). The prevalence rate of mild hyponatremia was 13.2% (95% CI: 10.1–16.3%) in patients without falls and 26.1% (95% CI: 19.8–32.4%) in patients admitted for falls. Mild hyponatremia was significantly associated with falls (P < 0.001) and the adjusted odds ratio (OR) was 3.02 (95% CI: 1.84–4.96).

**Conclusions:**

Because mild hyponatremia might be a risk factor for injurious falls and ED admission, determination of sodium levels during basic biomarker assessment on ED admission could be an important component of fall prevention strategies for the elderly.

## Background

Falls in the elderly is a major public health issue, as post-fall complications are the third leading cause of chronic disability and mortality [1]. Approximately 30% of community dwelling patients over 65 years of age, and half of patients over 80 years of age, fall at least once per year [2]. Furthermore, approximately 30% of falls result in moderate or severe injuries [3] and can affect the ability of an individual to engage in activities of daily living [4]. Therefore, falls may be considered as the first step towards dependence because approximately 40% of elderly people hospitalised for falls are institutionalised [5] and the mortality rate ranges from 7 to 11% [3, 5].

Thus, greater knowledge of risk factors of falls is extremely important for the prevention of accidents and dependence in the elderly. Falls have many risk factors. Internal risk factors include cognitive impairments, effects of medication, and sarcopenia; external risk factors include type of flooring and footwear [6]. Although the biologicals risk factors of falls remain poorly studied, hyponatremia is the most common electrolyte disorder in clinical practice [7]. This electrolyte imbalance is defined as a serum sodium concentration < 136 mEq/L [8] and can be divided into three stages: mild chronic hyponatremia (serum sodium of 130–135 mEq/)L, moderate hyponatremia (serum sodium levels of 125–129 mEq/L), and severe hyponatremia (serum sodium levels < 125 mEq/L [9];). Cross-sectional population studies have shown that the incidence of hyponatremia increases with age [10]. For example, hyponatremia affects approximately 18% of older people living in the community [7] and nearly 20% of elderly people who present to the emergency department (ED) [11].

Hyponatremia has many causative factors, especially in older patients with a greater predisposition due to physiological deterioration, comorbidities, and/or polypharmacy [12, 13]. The clinical symptoms are generally associated with the severity of hyponatremia, with the most frequently encountered symptoms being nausea, fatigue, and headache [8, 14]. Classically, mild chronic hyponatremia is defined as asymptomatic but it has recently been shown that it could be clinically significant [15], may be associated with the risk of fracture in ambulatory cohorts [16, 17], and could result in gait disturbances and impaired cognitive function [18]. Thus, the present study aimed to determine the prevalence of mild chronic hyponatremia, and to examine the relationship between mild chronic hyponatremia and falls, in an Emergency Geriatric Medicine Unit (MUPA Unit). It was hypothesised that mild hyponatremia would be a biological marker for fall risk in an elderly population.

## Methods

### Study design and setting

This study was an observational and cross-sectional investigation of patients ≥75 years of age at baseline, who had been admitted to the MUPA Unit at Limoges University Hospital Center (France) between November 1, 2014 and March 31, 2015. Patients who were living at home and admitted to the MUPA Unit for a non-vital emergency (i.e., not admitted for stroke or to the intensive cardiac unit or palliative unit) were included. The exclusion criteria included the absence of serum sodium measurements. Baseline demographic and clinical data were collected using a comprehensive geriatric assessment (CGA). Data confidentiality was ensured, written informed consent was obtained from each participant and the study protocol was approved by the local ethics committee. The study was conducted according to the principles of the Helsinki Declaration.

### Hyponatremia assessment

Hyponatremia was defined as a serum sodium level < 136 mEq/L, mild chronic hyponatremia as a serum sodium level between 130 and 135 mEq/L, moderate hyponatremia as a sodium level between 125 and 129 mEq/L, and severe hyponatremia as a serum sodium level < 125 mEq/L [9]. Data concerning natremia were obtained from the first blood test performed after the patient arrived at the MUPA Unit.

### Fall assessment

The incidence of falls was determined by reviewing medical records for falls documented either as part of the presenting complaint or during physical examination. A fall was defined as unintentionally coming to rest on the ground or other lower level not as a result of a major intrinsic event (e.g. myocardial infarction, stroke, or seizure) or an overwhelming external hazard (e.g. hit by a vehicle [19, 20];). All patients who reported falls were also asked to give the circumstances of the fall and report clinical outcomes, including whether any injuries (e.g. fractures), ED visits, and/or hospital visits had occurred. An injurious fall was identified using the question: “Did you hurt yourself in any way when you fell?”

### Other covariates

Relevant demographic, clinical, and outcome data were collected by a review of medical records and included the following variables: age; gender; frailty, assessed using the Short Emergency Geriatric Assessment (SEGA) scale [21]; autonomy, assessed using the Activity of Daily Living (ADL) scale [22]; medication adherence, assessed using the Morisky-Green test [23]; emergency frailty score, assessed using the Identification of Senior At Risk (ISAR) scale [24];; depression, assessed using the Geriatric Depression Scale (GDS) [25]; number of diseases; and number of prescription drugs used. All geriatric assessments were administered when the patient arrived at the MUPA Unit. The rates of rehospitalisation for falls and mortality at 1 month, 3 months, and 1 year were determined by a review of medical records.

### Data collection

Data were collected at baseline from the medical records of 696 patients who had been admitted to the MUPA Unit at Limoges University Hospital Center (France) between November 1, 2014 and March 31, 2015.

### Data analysis

Descriptive statistics are expressed as means ± standard error (SE). Student’s *t*-tests were used to compare the means of continuous, normally distributed data, to reveal factors associated with mild hyponatremia and falls. Univariate and multivariate analyses were conducted by applying a multiple logistic stepwise regression procedure [26] to obtain variables that were independently correlated with mild hyponatremia. All statistical tests were two-tailed and a significance level of *p* ≤ 0.05 was considered to indicate statistical significance. All analyses were performed with SAS® software (SAS Institute; Cary, NC USA).

## Results

### Participant characteristics

**Fig. 1:**
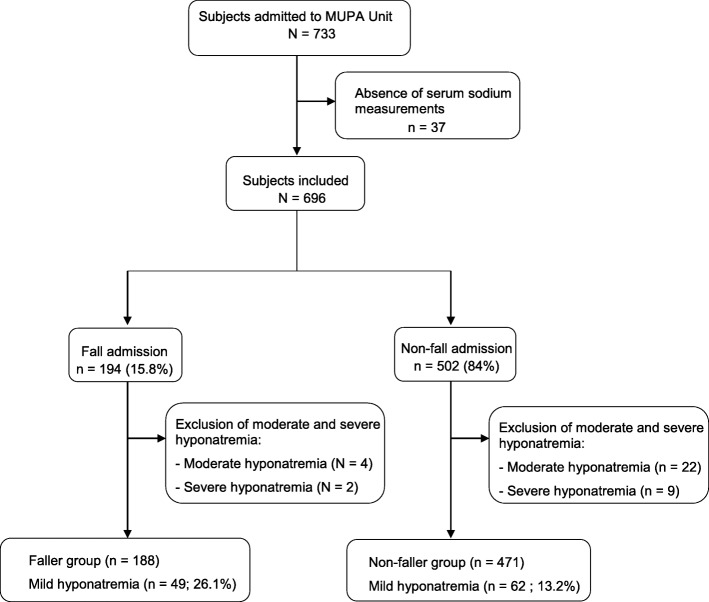
Flowchart of patients admitted to the Emergency Geriatric Medicine Unit (MUPA) Unit from November 1, 2014 to March 31, 2015 in Limoges, France

A total of 733 eligible patients were admitted to the MUPA Unit during the study period. Patients admitted to the MUPA Unit for a non-vital emergency (i.e., not for stroke), the intensive cardiac unit, or the palliative unit were included in the present study. Additionally, 37 patients were excluded due to the absence of serum sodium measurements in the first blood test conducted on arrival at the MUPA Unit; thus, a total of 696 patients were included in the final analysis (Fig. 1.)

The general characteristics of the study population are presented in Table 1; the mean age was 86.1 ± 5.6 years and the majority of patients were female (63.1%, *n* = 439). The study population was considered to be very frail based on the mean SEGA scale (13.4 ± 4) and ISAR scale (3.7 ± 1.2) scores. The mean ADL score was 3.9 ± 1.9, the Morisky-Greene test for medication adherence had a mean score of 3.5 ± 1.2, and the mean score on the GDS scale was 1.4 ± 1.4. Furthermore, the study population was polypathological (number of diseases per patient = 4.3 ± 2.6) and polymedicated (mean number of drugs used = 6.4 ± 3.1).

**Table 1:** Characteristics of patients admitted to the MUPA Unit

Variables	Patients (*n* = 696)
Age (y, mean ± SD)	86.1 ± 5.6
Female n (%)	439 (63.1)
Frailty evaluation – SEGA (mean ± SD)	13.5 ± 4
Not particularly frail (SEGA ≤8), n (%)	70 (10.0)
Frail (8 < SEGA ≤11), n (%)	93 (13.4)
Very frail (SEGA > 11), n (%)	389 (55.9)
Unknown, n (%)	144 (20.7)
Autonomy evaluation – ADL (mean ± SD)	3.9 ± 1.9
Medication adherence – Morisky Test (mean ± SD)	3.5 ± 1
Emergency frailty score – ISAR (mean ± SD)	3.7 ± 1.2
Subjects not at risk (ISAR < 2), n (%)	13 (1.9)
Subjects at risk (ISAR ≥2), n (%)	368 (52.9)
Unknown, n (%)	315 (45.2)
Geriatric Depression Scale – GDS (mean ± SD)	1.4 ± 1.4
No depression (GDS = 0), *n* = 146	146 (21.0)
Depression (GDS ≥ 1), *n* = 200	200 (28.7)
Unknown, *n* = 350	350 (50.3)
Number of diseases (mean ± SD)	4.3 ± 2.6
Number of drugs (mean ± SD)	6.4 ± 3.1

### Prevalence of hyponatremia

Hyponatremia (< 136 mEq/L) was detected in 148 subjects and the prevalence was 21.30% (95% confidence interval [CI]: 18.3–24.3%, serum sodium concentration = 130.8 ± 6.3 mEq/L). Of the 696 subjects, 15.9% (*n* = 111; 95% CI: 13.2–18.6%) had mild chronic hyponatremia, 3.7% (*n* = 26; 95% CI: 2.3–5.1%) had moderate hyponatremia, and 1.6% (n = 11; 95% CI: 0.7–2.5%) had severe hyponatremia.

### Mild hyponatremia and its association with falls

Table 2 compares baseline characteristics between patients admitted for a fall and those not admitted for a fall. Patients with moderate or severe hyponatremia (*n* = 37) were not included and data regarding general characteristics, geriatric assessments, comorbidities, and drugs at baseline were compared. Patients admitted to the MUPA Unit for a fall were significantly less dependent than those admitted for other reasons (ADL score: 4.6 ± 1.5 vs. 3.6 ± 2.0, respectively, *p* < 0.0001); patients admitted for a fall were also less polypathological (number of diseases: 2.5 ± 2.0 vs. 3.2 ± 2.1, *p* = 0.0008). The prevalence of mild hyponatremia was 13.2% (95% CI: 10.1–16.3%) in patients without falls and 26.1% (95% CI: 19.8–32.4%).

**Table 2:** Characteristics of patients admitted to the MUPA Unit for falls

Variables	Falls (*n* = 188)	Non-falls (*n* = 471)	*P*-value
Age (y, mean ± SD)	87 ± 5.9	87 ± 5.6	0.73
Female n (%)	122 (64.9)	287 (60.9)	0.34
Frailty evaluation – SEGA (mean ± SD)	13.1 ± 3.5	13.7 ± 4.2	0.07
Little frail (SEGA ≤8), n (%)	19 (10.1)	48 (10.2)	0.66
Frail (8 < SEGA ≤11), n (%)	30 (16.0)	57 (12.1)	0.26
Very Frail (SEGA > 11), n (%)	109 (57.9)	262 (55.6)	0.77
Unknown, n (%)	30 (16.0)	104 (22.1)	0.17
Autonomy evaluation – ADL (mean ± SD)	4.6 ± 1.5	3.6 ± 2.0	< 0.0001
Morisky (mean ± SD)	3.3 ± 1.1	3.6 ± 1.1	0.08
Emergency frailty score – ISAR (mean ± SD)	3.8 ± 1.0	3.8 ± 1.2	0.83
Subjects not at risk (ISAR < 2), n (%)	1 (0.5)	10 (2.1)	0.19
Subjects at risk (ISAR ≥2), n (%)	112 (59.6)	237 (50.3)	0.25
Unknown, n (%)	75 (39.9)	224 (47.6)	0.27
Geriatric depression scale – GDS (mean ± SD)	1.2 ± 1.4	1.5 ± 1.5	0.18
No depression (GDS = 0), n (%)	51 (27.1)	87 (18.5)	0.05
Depression (GDS ≥ 1), n (%)	56 (29.8)	132 (28.0)	0.78
Unknown, n (%)	81 (43.1)	252 (53.5)	0.27
Number of diseases (mean ± SD)	2.5 ± 2.0	3.2 ± 2.1	0.0008
Number of drugs (mean ± SD)	6.0 ± 3.0	6.6 ± 3.1	0.05

Student’s t tests were used for comparisons of quantitative variables and Fisher’s exact tests were used for comparisons of qualitative variables between groups

in patients admitted for falls. Mild hyponatremia was significantly associated with falls (P < 0.001) and the adjusted odds ratio (OR) was 3.02 (95% CI: 1.84–4.96). The association between mild hyponatremia and falls remained significant after controlling for covariates (Table 3).

**Table 3:** Prevalence rates of mild hyponatremia in patients admitted for falls and control subjects

Variable	Patients (*n* = 188)	Controls (*n* = 471)	Unadjusted		Adjusted	
	Percent (number)		*OR*	*P*-value	*OR*	*P*-value
Mild hyponatremia	26.1 (49)	13.2 (62)	2.33 (1.53–3.55)	0.001	3.02 (1.84–4.96)	0.001

Adjusted for age, sex, frailty status, functional autonomy, cognitive and depression status, number of drugs, and comorbidity index

## Discussion

### Main results

The present population of patients admitted to the MUPA Unit was very frail and polypathological and the prevalence of mild hyponatremia was 15.9% (95% CI: 13.2–18.6%). The rate of fall recurrence was higher in females with mild hyponatremia than in males; importantly, the females were older and frailer than the males. There was a significant association between mild hyponatremia and the risk of injurious falls (falls resulting in admission to the MUPA Unit) but there were no significant associations among the risk of readmission for injurious fall, mortality, and level of natremia.

### Study strengths

The present study is the first to demonstrate a link between mild hyponatremia and injurious falls among patients admitted to the MUPA Unit. Renneboog and al [18]. and Gunathilake and al [26]. also observed this association but investigated patients from a classic ED and living in the community but did not focus on geriatric assessments. The present study assessed the relationship between and falls among patients in the MUPA Unit and performed specific geriatric assessments. The relationship between natremia and falls is very interesting, particularly in the context of patients from a MUPA Unit, because mild hyponatremia is highly prevalent and these patients have a greater risk of falling. The present study is the first to investigate this particular relationship and, notably, revealed a significant difference between males and females with respect to the link between mild hyponatremia and risk of falls. Females admitted to the MUPA Unit were older and more dependent than the males, which may explain why their risk of falls was higher. These results demonstrate the necessity for the implementation of falls prevention strategies in the ED.

### Study limitations

The present study was limited in that it focused on serum sodium concentrations obtained during the first blood test on arrival at the MUPA Unit. Thus, it was not possible to determine if the hyponatremia was acute or chronic because ofthe absence of longitudinal data. So, we concluded that “mild hyponatremia” was associated with falls in older adults admitted to the MUPA Unit. Further prospective and longitudinal studies of this relationship should be conducted. Additionally, the present study population was highly selective, in that the patients were admitted to the MUPA Unit for non-vital emergencies and the study focused only on geriatric assessments.

### Study context and implications

The prevalence of hyponatremia in the present study (21.3%) was slightly higher than that reported by previous studies. For example, Upadhay et al. [7] reported a prevalence of 18%. However, this study recruited patients admitted to the MUPA unit whereas the participants lived at home in the previous study. Hyponatremia is associated with the occurrence of geriatric disorders, such as attentional and postural disorders, leads to a significant increase in the risk of fractures, and may be a risk factor of osteoporosis [18, 27, 28]. Some studies have implicated hyponatremia, particularly mild chronic hyponatremia, in cognitive disorders and suggested that chronic hyponatremia could induce attention disorders [18].

Additionally, patients with mild chronic hyponatremia may be more likely to develop osteoporosis [27] and this disorder could increase the risk of a fracture [16, 29] and osteoporosis [30]. The present study observed an association between mild chronic hyponatremia and falls among patients in the MUPA Unit, whereas previous studies included patients from classic EDs [18, 31, 32] and home-based populations [17]. Ahamed et al. [33] observed the same association between hyponatremia and falls but reported that the risk of falls in patients with mild hyponatremia was similar to the risks in those with moderate to severe hyponatremia.

## Conclusion

In conclusion, the present findings demonstrated that elderly patients admitted to the MUPA Unit, and who exhibited mild chronic hyponatremia during the first blood test following arrival, had a higher risk of falling. Mild chronic hyponatremia could be considered a risk factor for falls and, thus, determination of sodium levels in ED assessments might be an important component of strategies for preventing injurious falls in the elderly. Nonetheless, the present findings of an association between mild chronic hyponatremia and falls should be confirmed by longitudinal and prospective studies.

## Data Availability

the dataset is not available but can be requested from the corresponding author.

## Abbreviations

ADL: Activity of Daily Living
CGA: Comprehensive Geriatric Assessment
CI: Confidence Interval
ED: Emergency Department
GDS: Geriatric Depression Scale
MUPA: Médecine d’Urgence de la Personne Agée
OR: Odd Ratio
SEGA: Short Emergency Geriatric Assessment
